# Well-Being of Teachers: The Role of Efficacy of Teachers and Academic Optimism

**DOI:** 10.3389/fpsyg.2021.831972

**Published:** 2022-01-26

**Authors:** Kuo Song

**Affiliations:** Center for Faculty Development, Harbin University, Harbin, China

**Keywords:** academic optimism, teachers’ efficacy, teachers’ well-being, positive psychology, societal theory

## Abstract

In previous decades, the well-being of teachers has been at the center of the attention of researchers in several practical investigations on various subjects such as language learning. The objective of this review is to clarify this construct and add new information on the predominance of the well-being of teachers and organize factors impacting it. Nevertheless, among factors influencing the levels of well-being, the focus of this review is on two constructs, namely, optimism “as a new concept in positive psychology,” societal theory, and collective school assets in education, and efficacy of teachers as an individual source in teachers. This review tries to focus on the eminent role of teacher efficacy and optimism in the process of education and also clarify their consequences on the well-being of teachers. In a nutshell, this review can provide implications for language teaching participants in the academic context.

## Introduction

Education can be a rewarding career with significant, impactful, and crucial work; nonetheless, the intricacy of its quality can make it difficult. In fact, up to one-third of educators are anxious, and a similar number of educators quit their careers in the initial 5 years of education (Ingersoll and May, 2012). Education is a highly emotional career, connected to prominent levels of anxiety, and can lead to work dissatisfaction, mental illness, and diminished well-being, so scholars, managers, and policymakers are becoming increasingly concerned about the well-being of educators because of these worrisome patterns (Keller et al., 2014). Even though a large amount of literature highlights the anxiety-provoking quality of the teaching career and numerous consequences of anxiety, there is not enough knowledge of what makes educators’ capability of flourishing in the workplace easier (Jennings and Greenberg, 2009). On account of these developments, students, managers, and policymakers have become more involved in the well-being of teachers (Collie et al., 2012). Research evidence shows that the well-being of educators is a crucial element in educator effectiveness, educator retention, and the well-being of learners they instruct. Therefore, enhancing well-being among teachers has advantages both for themselves and the success of an institution (Giorgi et al., 2017). Hence, there has been a growing interest in searching for protective elements and interferences that advance well-being in the professional context (Shropshire and Kadlec, 2015). A further line of the study stated that a significant predictor of well-being and prospering is the stability between positive and negative concerns (Fredrickson and Losada, 2005). In the literature on educators, well-being has been studied primarily with focus on deconstructive emotions of stress and burnout (Spilt et al., 2011). In addition, several issues were promoted in past studies with the objective of advancing a greater constructive viewpoint, and pertinent concepts studied encompass educator self-efficacy and academic optimism (Orkibi and Ronen, 2017; Han and Wang, 2021).

Well-being can be analyzed from the viewpoint of personal depths and complexities that are the essence of positive psychology (PP; Wang et al., 2021). Focusing on human forces takes constructive quality into well-being at work. Seligman and Peterson (2003) have explored human assets and how people can accomplish positivity in different life circumstances, courageousness, thoughtful for others, fairness, self-regulation, optimism, and hope, among other things, assets through which individuals can comprehend and attain well-being in life and at work. A singular distinctive factor that displays the degree to which people possess generalized positive anticipations for their future is called optimism (Carver et al., 2010). As it builds the bravery to fulfill the demanding situations of labor and family life, it is a crucial requirement for individual and expert achievement. Moreover, optimism advances one’s capability of producing innovative solutions to troubles faced in the workplace (Akhtar and Saleem, 2020). In addition, PP also represents keystones or causes of educational optimism, and academic optimism is further defined as being related to PP (Gürol and Kerimgil, 2010).

Alternatively, the review of related literature has presented a robust mark that self-efficacy is maintained to be a constructive characteristic that needs to be examined in scholastic studies, and that it is characterized as one’s judgment regarding capability and competence to deal with various circumstances and carry out a particular assignment (Hoy and Tarter, 2011; Han and Wang, 2021). The social cognitive theory highlights the important function that self-efficacy performs on human behavior, as self-efficacy is not completely connected to one’s conviction regarding their capabilities. Indeed, it is one’s conviction regarding the accomplishment of an assignment based on their beliefs in that issue (Sezgin and Erdogan, 2015). Judgments of educators regarding their ability to affect learners’ engagement and education are known as teacher efficacy (Fathi et al., 2021). In the last 20 years, some researchers have mentioned that educator efficacy is affected by educators’ awareness of their specific educational context, evaluations of assets and support given to them, and requirements of their instructing assignments (Tschannen-Moran and Hoy, 2001). Therefore, teacher self-efficacy is a necessary consideration in influencing educators’ view of their job, their classroom events, and their impact on scholars’ conclusions (Tschannen-Moran and Hoy, 2001). Personal resources such as self-efficacy and optimism were found to mediate the relationship between job resources and commitment (Xanthopoulou et al., 2007), and to comprehend the consequence of issues that are focused on teacher well-being, this study tries to scrutinize the position of the above-mentioned perceptions in language teacher well-being.

## Review of the Literature

### Optimism

Optimism is generalized constructive anticipation that good occurrences will take place in place of bad ones, causing optimists to deal with life’s difficulties with self-confidence in place of doubt (Carver et al., 2010). Optimism alludes to a viewpoint on life and presents a circumstantial format for managing traumas and guides reactions and intellectual frame of anxiety-provoking occurrences. It has been hypothesized as a descriptive style; a way circumstances may be delineated. In an optimistic descriptive style, deconstructive circumstances have much less impact on prospective perception and practice, and constructive circumstances may be more inspiring for prospective practices (Meevissen et al., 2011). Academic optimism contains three assets, namely, academic emphasis, cooperative efficacy, and staff trust in learners, and it works in all school stages and is initially speculated and demonstrated at the beginner level (Hoy and Tarter, 2011). This concept is hypothesized as teachers’ confidence that they can affect learners’ achievement by highlighting academics, believing in their own capabilities to endure in case of facing problems, and by presuming learners to work together in this course of action (Beard et al., 2010).

### Self-Efficacy

Self-efficacy is a predominant topic in describing how a person acts, feels, and responds when confronting stressful conditions (Downes et al., 2017; Han and Wang, 2021), and it is critical in developing the performance of student to make educational progression easier (Van Dinther et al., 2011). Teacher self-efficacy is a motivational conception to signify the insight and faith of educators to do teaching assignments (Moulding et al., 2014). This concept is taken from the intellectual societal theory that underlines the value of social practice and need for learning in the development of fostering a person’s behavior (Mahler et al., 2018). In relation to this scheme, one’s preference in a particular condition is based on his own observation that shapes his intellectual development and societal manners in forthcoming experiences, and the collaboration of cognitive, interactive, and ecological aspects affect the activity of a person (Qureshi, 2015). Experience, belief, and mindset are among cognitive aspects, while ecological factors connect with social standards, community contact, and impact on others (Mahler et al., 2018). Self-efficacy has additionally been shown to have an instantaneous effect on performance, which correlates with overall ability. Educators who possess excessive degrees of self-efficacy frequently undertake appropriate academic strategies and methods, bringing about improved learner participation and academic achievement (Ipek et al., 2018).

### Well-Being

Well-being is defined from two perspectives, the hedonic and eudemonic types (Ryan and Deci, 2001). The former view is only related to pleasure and life enjoyment and describes well-being as acquiring happiness and evading agony. The major aim of the hedonic approach is to increase contentment, shaping what is to be identified as “subjective wellbeing” (Kim-Prieto et al., 2005). Alternatively, the latter approach to well-being is focused on self-actualization and involves the nature of individual life and fulfillment of abilities. This perspective supports the foundation for psychological well-being and takes advantage of these capabilities to utilize assets in a way that they can give sense to lifetime (Mercer and Gregersen, 2020; Han, 2021a). Indeed, these are fundamental approaches indicative of well-being theory that emphasizes a long-term outlook on meaning and commitment to happiness, instead of short-term hedonistic viewpoints of enjoyment and pleasure. It means that a person is flourishing, or indeed undergoing great well-being when they encounter regular positive emotions, are engaged in various fields in life, have relationships with other individuals, and achieve their objectives (Hessel et al., 2020; Wang and Guan, 2020).

## Implications and Future Directions

Regarding practical implications, this research highlights the position of frequent particular and related aspects that can be spoken clearly in teacher teaching programs to make teachers ready. Indeed, they should be prepared to find ways to cope with challenges in the process of their teaching career and to authorize them with capabilities to be more effective and successful in their occupation. Teacher efficacy should be viewed as an important matter for cultivating teacher well-being. This study revealed that teacher efficacy could encourage the effect of job resources on teacher well-being by enhancing their efficacy and level of optimism. Therefore, academic stakeholders are required to draw their attention to the plan and application of efficient teaching staff training programs. Moreover, by boosting optimism in teacher training programs, comprehensive instructive schemes should be conducted in a way to improve the level of well-being of novice and professional teachers. It also lessens their stress and certifies them to feel pleased and to remain engaged in their career.

The teachers with the more intellectual optimistic level are more likely to define the direction as delivered in their colleges in a planned design, which concentrates on the aspirations of the college. Truly, optimists are more inclined to take advantage of dynamic coping approaches and keep their work.

Teacher educators should try to address teacher wellbeing; they could assist them to manage their lessons and preliminary teaching practices, and provide them with tactics to thrive in their career in the long run (Birchinall et al., 2019; Han, 2021b). They should emphasize more on types of issues that encourage constructive emotions and adjust to negative ones in case of tension. The results of this review highlight the status of tackling positive qualities, namely, optimism and efficacy, in intervention procedures that subsequently could support teachers to enhance their well-being. When language teachers attain their well-being, they will enthusiastically expand their creativity to present activities and tasks in the classroom, and their presentations, likewise, influence the success of their learners. Moreover, educators and learners will attain their learning goals when the process of learning is interesting and the interpretation of condition demonstrates that teachers’ well-being has been related to positive students’ learning outcomes (Baumeister et al., 2003). Teachers’ optimism is significant because they are regarded as the role patterns for learners and their opinions have a considerable role in their life cycles. The optimistic behavior of teachers can enhance positivity in learners, and it may assist them in looking at a different side of an issue even during demanding conditions and dealing with them efficiently (Hasnain, 2014). Further inquiries are suggested to be carried out with the aim of focusing more on other types of constructive feelings, for example, gratefulness, empathy, self-care, tolerance, hope, and enjoyment on the one hand, and on the other hand, destructive types of feeling, such as boredom and resentment. In a nutshell, empirical studies should be conducted in order to guarantee the mediator role of the variables of the study such as teacher optimism and self-efficacy on teacher well-being, and similar studies can be conducted to consider the same issue by focusing on learners.

## Author Contributions

The author confirms being the sole contributor of this work and has approved it for publication.

## Conflict of Interest

The author declares that the research was conducted in the absence of any commercial or financial relationships that could be construed as a potential conflict of interest.

## Publisher’s Note

All claims expressed in this article are solely those of the authors and do not necessarily represent those of their affiliated organizations, or those of the publisher, the editors and the reviewers. Any product that may be evaluated in this article, or claim that may be made by its manufacturer, is not guaranteed or endorsed by the publisher.
